# Multiple Fungi May Connect the Roots of an Orchid (*Cypripedium reginae*) and Ash (*Fraxinus nigra*) in Western Newfoundland

**DOI:** 10.3389/ffunb.2022.805127

**Published:** 2022-03-01

**Authors:** Nimalka M. Weerasuriya, Katarina Kukolj, Rebecca Spencer, Dmitry Sveshnikov, R. Greg Thorn

**Affiliations:** ^1^Department of Biology, University of Western Ontario, London, ON, Canada; ^2^School of Science and the Environment, Grenfell Campus, Memorial University, Corner Brook, NL, Canada

**Keywords:** showy lady's slipper orchid, black ash, mycorrhiza, metabarcoding, Illumina MiSeq, mixotrophy, common mycorrhizal network, ITS2

## Abstract

Showy lady's slipper (*Cypripedium reginae* Walter, Orchidaceae) and black ash (*Fraxinus nigra* Marshall, Oleaceae) often co-occur in close proximity in fens in western Newfoundland, Canada. Metabarcoding of DNA extracted from root samples of both species following surface sterilization, and others without surface sterilization was used to determine if there were shared fungal endophytes in the roots of both species that could form a common mycorrhizal network between them. A wide variety of fungi were recovered from primers amplifying the nuclear ribosomal internal transcribed spacer region (ITS2). Sixty-six fungal sequences were shared by surface-sterilized roots of both orchid and ash, among them arbuscular mycorrhizal fungi (*Claroideoglomus, Dominikia, Glomus* and *Rhizophagus*), ectomycorrhizal fungi (*Inocybe* and *Tomentella*), the broad-host root endophyte *Cadophora orchidicola*, along with root pathogens (*Dactylonectria, Ilyonectria, Pyricularia*, and *Xylomyces*) and fungi of unknown function. There appear to be multiple fungi that could form a common mycorrhizal network between *C. reginae* and *F. nigra*, which might explain their frequent co-occurrence. Transfer of nutrients or carbon between the orchid and ash via one or more of the shared fungal endophytes remains to be demonstrated.

## Introduction

Orchids (Orchidaceae) are famous for their dependence on fungi. The tiny “dust seeds” of most orchids need to be penetrated, colonized and fed by a compatible fungus to initiate germination, and most orchids remain nutritionally dependent on their associated fungus while the seedling grows into a protocorm, a nutritional mode known as mycoheterotrophy (Burgeff, 1959; Rasmussen and Rasmussen, 2009). While achlorophyllous plants, including orchids, need an external source of energy throughout their life, the extent of mixotrophy, or partial nutritional dependence on fungi, in chlorophyllous orchids has been little investigated. Substantial evidence has accumulated to show that several Orchidaceae species that are chlorophyllous and photosynthetic as adults continue to obtain at least a part of their carbon energy from a shared, or common mycorrhizal network; Temperate orchid genera in which this has been documented include *Cephalanthera, Epipactis* (Bidartondo et al., 2004), *Goodyera* (Voronina et al., 2018), *Listera* (Gebauer and Meyer, 2003), *Ophrys* (Girlanda et al., 2011), and *Rhizanthella* (Warcup, 1985). Where the association is known, it is usually with ectomycorrhizal (ECM) fungi (Selosse et al., 2006), or with known root pathotrophic (Peschke and Volz, 1978; Vujanovic et al., 2000) or saprotrophic endophytes (Wang et al., 2021). Arbuscular mycorrhizal fungi (AMF) have been found associated with the roots of the Mediterranean grassland orchids *Anacamptis* and *Ophrys* (Voyron et al., 2017), and two temperate North American species of the lady's slipper orchids, *Cypripedium californicum* and *C. parviflorum* (Shefferson et al., 2005). However, the role of AMF in orchid nutrition has not been experimentally determined.

**Figure 1:**
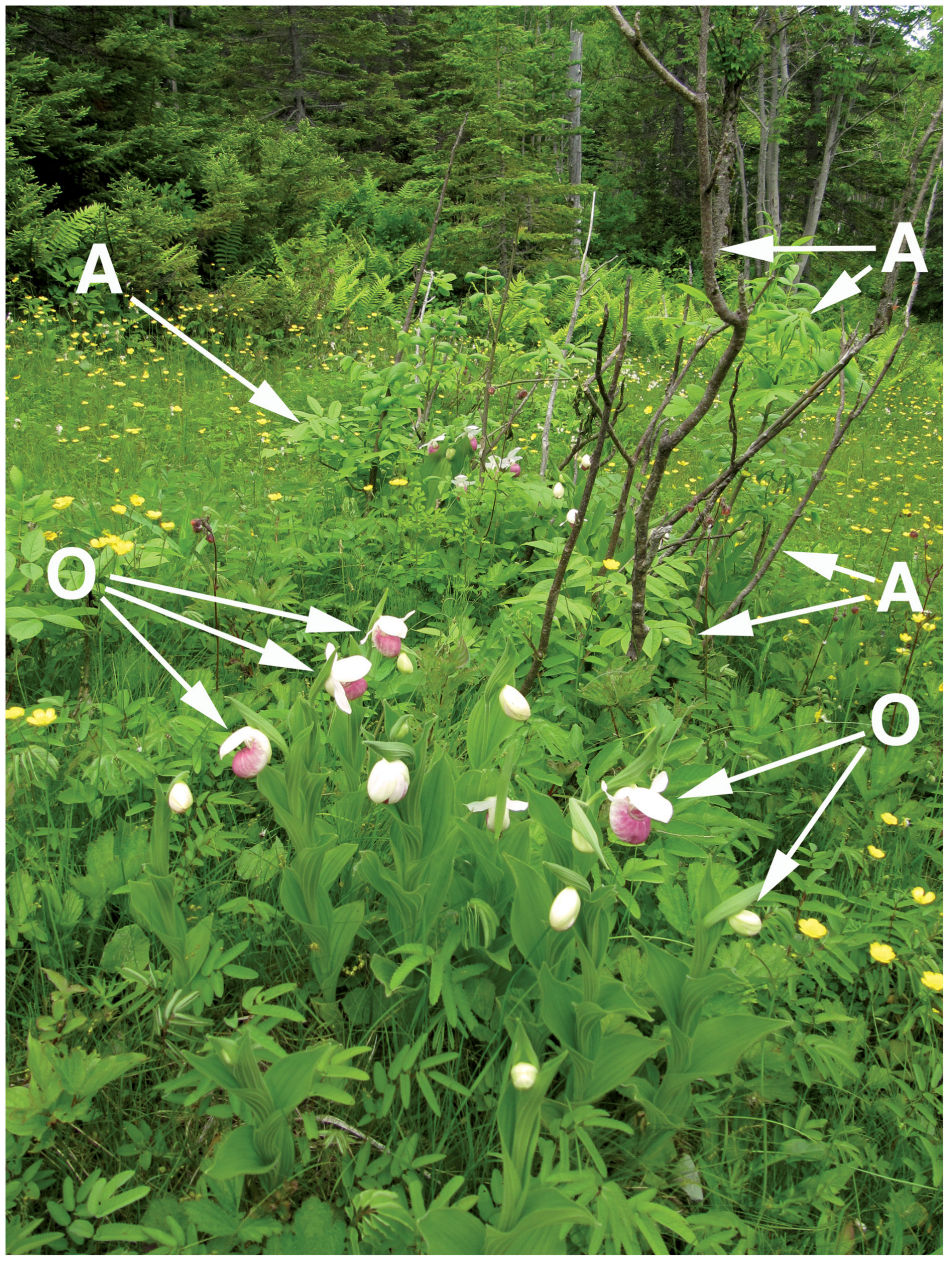
A sampling site in a Newfoundland fen, with showy lady's slipper (*Cypripedium reginae*; arrowheads labeled O) surrounding a shrubby growth of black ash (*Fraxinus nigra*; arrows labeled A). Photograph by Voitk (2011).

On the island of Newfoundland, Canada, the showy lady's slipper (*Cypripedium reginae* Walter) is frequently found growing near small trees of black ash (*Fraxinus nigra* Marshall) (Voitk, 2011). Since both plants are locally uncommon and at the edge of their geographic ranges, the pattern of the orchids forming a circle within 2 m of the base of the ash (Figure 1) seemed unlikely to be due to chance or a shared habitat preference, although both grow in wetland habitats (Wright and Rauscher, 1990; Kennedy and Walker, 2007). The mycorrhizal status of *C. reginae* is poorly known (Curtis, 1939; Zelmer et al., 1996), but the main mycorrhizal partners of seven other species of *Cypripedium* are members of Tulasnellaceae (Basidiomycota, Agaricomycetes) (Shefferson et al., 2005), a group that also form ECM (Tedersoo and Smith, 2013). In contrast, *F. nigra* roots have been shown to have associations with arbuscular mycorrhizal fungi, with no evidence of ECM associations (Malloch and Malloch, 1982; Brundrett et al., 1990). The objective of our study was to analyze and compare the fungi associated with the roots of *C. reginae* and *F. nigra*, growing separately or together in sites in western Newfoundland, to determine if these plants share any fungi that could form a common mycorrhizal network, which, in turn, taken together with the showy lady's slipper's mixotrophic lifestyle, might explain its frequent co-occurrence with black ash.

## Materials and Methods

### Sample Collection

**Figure 2:**
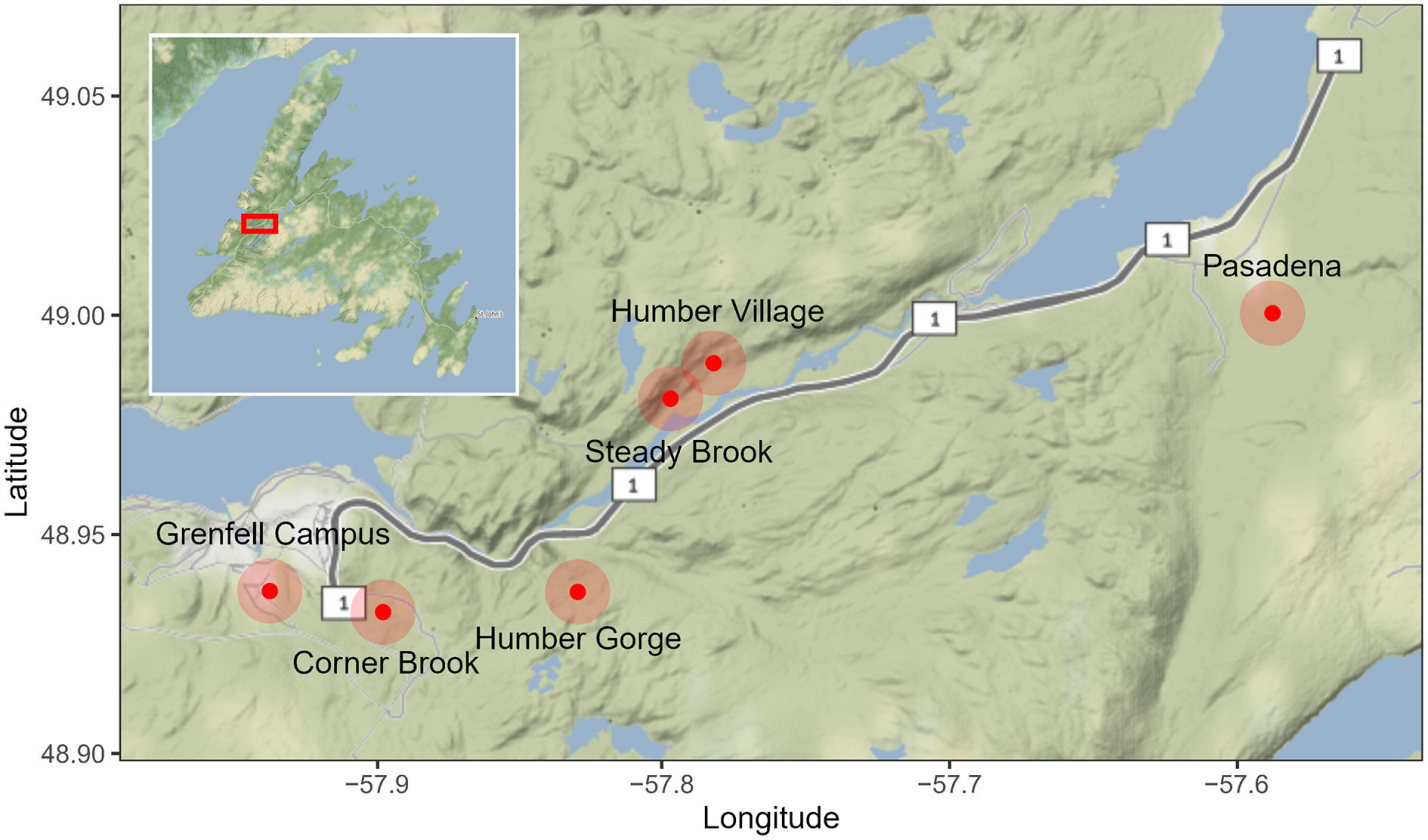
Approximate sampling locations in Western Newfoundland, Canada. Samples from a total of 54 plants were collected, 27 showy lady's slipper orchid (*Cypripedium reginae*) root samples and 27 black ash (*Fraxinus nigra*) root samples. Basemap provided by Google Maps through the ggmap package in R.

Roots of *Cypripedium reginae* and *Fraxinus nigra* were collected between 29 July and 18 August 2019, from six locations in western Newfoundland: Grenfell Campus fen, Corner Brook fen, Humber Gorge, Steady Brook, Humber Village, and Pasadena (Figure 2). The three westernmost sites are closer to Marble Mountain, with more rocky, calcareous soils, whereas the easternmost sites were in forest-surrounded fens and ditches. Samples were labeled with plant name (*F. nigra* or *C. reginae*), and presence or absence of the other plant growing within 15 m, yielding four comparison groups: (a) orchids with no ash nearby (ONA), (b) orchids with ash nearby (AO-Orch), (c) ash with no orchids nearby (ANO), and (d) ash with orchids nearby (AO-Ash). A total of 54 root samples (27 each of *F. nigra* and *C. reginae*) were collected (each approximately 10 root tips, 2–8 cm per sample, one sample per plant), rinsed with water, and immediately divided, with five roots from each sample preserved in CTAB (cetyl trimethyl ammonium bromide, for DNA extraction) (Gardes and Bruns, 1993) and the other five in FAA (formalin-acetic acid-alcohol, for microscopy) (Supplementary Table 1). All orchid and ash plants survived the sampling, confirmed in site visits the following year.

### Microscopy

Root samples were cleared and stained for microscopy (Brundrett et al., 1994). In short, thicker orchid roots were sectioned transversely and longitudinally, and stained using trypan blue (0.01% w/v) in lactophenol for 1 min, followed by mounting using lactoglycerol (1:2:1 lactic acid, glycerol and water). Whole ash roots were cleared with 10% KOH (w/v) at 80°C overnight (~12 h), rinsed, and stained as above. Samples were examined and photographed with a ZeissZ1 microscope with brightfield illumination (Biotron, University of Western Ontario).

### Surface Sterilization and DNA Extraction

Approximately half of the CTAB root samples were randomly assigned to a surface sterilization protocol to denature the DNA of organisms on the outsides of the roots to compare endophytic fungi to non-surface-sterilized samples that included root-associated rhizosphere fungi (Supplementary Table 1). Briefly, 100 mg of 0.5–1 cm segments of root tips were cleaned by vortexing in 0.1 M sodium pyrophosphate (60 s), 100% ethanol (molecular grade; 5 s), 0.5% sodium hypochlorite (freshly diluted household bleach, 60 s), 70% ethanol (60 s), followed by three rinses in sterile molecular grade water. Samples that were not surface-sterilized were only rinsed with sodium pyrophosphate and sterile water. All root tips were plunged into and dabbed on malt extract agar (MEA) with chloramphenicol (100 μg/mL) prior to DNA extraction to assess the presence of culturable fungi on their surfaces. Plates were incubated in the dark at room temperature for 2 weeks to record any fungal growth. Extraction of DNA from root tips of *Cypripedium reginae* and *Fraxinus nigra* was conducted using a bead beating protocol from the Quick-DNA ™Plant/Seed Miniprep kit (Zymo Research, Irvine, California, United States). Extracts were quantified with a Thermo Scientific Nanodrop2000 Spectrophotometer and stored at −20°C until PCR amplification.

### PCR Amplification

Each DNA sample was PCR-amplified using primers 5.8S-Fun and ITS4-Fun that amplify the ITS2 region of rDNA from most fungi (Taylor et al., 2016). The variability due to evolutionary divergence found at this locus is, in many fungi, adequate to enable identification of nucleotide sequences to the level of species (Taylor et al., 2016). Both primers were modified for Illumina MiSeq sequencing by including a forward or reverse Illumina adapter, a 4 base pair linker (NNNN), and an 8-nucleotide index barcode that allows sequences to be assigned to sample origin after multiplexing. The PCR mix included Toughmix (Quanta Biosciences) polymerase master mix with 50 × loading dye, primers, and 2 μL of each sample DNA and were run in a hot-start (2 min at 94°C), 30-cycle program of 94°C for 30 s, 55°C for 30 s, and 72°C for 30 s, yielding amplicons of approximately 380 bp in length, including the adapters, barcodes, and linkers. One negative control was used (sterile molecular grade water) and two positive controls of DNA extracts from *Saccharomyces cerevisiae* (Ascomycota) and *Agaricus bisporus* (Basidiomycota). Sequences were obtained using a 2 × 300 kit on an Illumina MiSeq sequencer at Robarts Research Institute, London, ON.

### Illumina Sequencing and Data Analysis

Amplicons were initially demultiplexed using a custom BASH script (https://github.com/nweerasu/primer_pull). Forward and reverse FASTQ files for each primer were again demultiplexed following a Python script (https://github.com/ggloor/miseq_bin/blob/master/demultiplex_dada2.pl) to separate samples, where primers and barcode sequences were removed. Sample FASTQs were processed through DADA2 (Callahan et al., 2016), following a combination of scripts including the DADA2 workflow for Big Data: Paired-end (1.4 or later) (https://benjjneb.github.io/dada2/bigdata_paired.html) and a modified DADA2 pipeline for paired-end sequences that provided summary statistics (https://github.com/ggloor/miseq_bin/dada2_workflow_1.4.R). Taxonomic assignments used the UNITE ITS General FASTA release (v 8.3) using singletons set as RefS (Abarenkov et al., 2021). Parameters for filtration, trimming, merging, and chimera checking steps within DADA2 are provided in Supplementary Table 2. ITS2 data were additionally filtered to remove singletons and minimize sample bleeding using both positive controls (*S. cerevisiae* and *A. bisporus*) as guides. A minimum read threshold of ≥10 reads and a minimum relative abundance threshold of ≥0.03% in each sample was used to reduce index-hopping, or sample bleeding, of amplicon sequence variants (ASVs) based on our positive and negative controls.

Taxonomy was validated using reference sequences downloaded from BLASTn and included with the ASVs in a neighbor-joining tree built with a MAFFT Online alignment (Kuraku et al., 2013; Katoh et al., 2018). Taxonomic clades were created for heatmaps and network analysis to label ASVs from the same individual. Maximum-Likelihood trees of shared orchid/ash ASVs found in SS samples were created and ASVs were grouped into clades where distances between nodes were <0.01. Non-target sequences were filtered from each ASV table prior to analysis. Nomenclature follows IndexFungorum.org.

### Statistical Analyses and Visualization

The packages ggmap, microeco, ggplot2, ggpubr, rstatix, and vegan were used for analyses and visualization of community similarities and differences within R (v 4.1.1) (Kahle and Wickham, 2013; Wickham, 2016; Kassambara, 2020, 2021; Oksanen et al., 2020; Liu et al., 2021). Alpha diversity analyses were done by calculating all applicable diversity indices: observed richness, Shannon's index H′, Simpson diversity index, inverse Simpson diversity index, and Fisher's alpha index for each group of samples (ONA, ANO, AO-Ash, and AO-Orch), split by surface sterilization (sterilized—SS, not sterilized—NSS), and sampling location (six levels, see Methods). An ANOVA and Duncan's Multiple Range Test (DMRT; agricolae package) (De Mendiburu, 2021) determined whether observed differences of the group means were significant, and which groups were significantly different for each diversity measure.

ANOSIM (vegan) with 999 permutations was used to detect any differences in ASV composition between samples by comparing the hosts (orchid or ash; presence or absence of the other host nearby), differences in surface sterilization, sampling location, and any batch differences between DNA extraction/PCA protocols (An et al., 2019). All comparisons used a Bray-Curtis distance matrix with a Kruskal-Wallis test for group comparisons, with between-group comparisons made using Dunn's Test for multiple comparisons with Benjamini-Hotchberg adjusted *p*-values.

The significant orchid-ash presence and location factors were visualized using PCoA (Bray-Curtis distance) and NMDS ordinations. The most relevant ASVs are included as vectors for each plot type, with the top 10 ASVs that have the highest correlation to the sample matrix included in the PCoA plots, and the most significant 10 ASVs (*p* < 0.05) included in the NMDS plots.

Prior to network analysis, 66 ASVs that were found in both orchid and ash roots were clustered into 45 clades (distance <0.01) in a ML tree using MEGA X (Kumar et al., 2018; Stecher et al., 2020). Network analyses were done using a non-parametric Spearman's correlation matrix. The correlation optimization parameter (COR_optimization) in the microeco package was used, where the Random Matrix Theory is applied to select the optimal correlation cutoff for the data set (Deng et al., 2012; Liu et al., 2021). Network files were exported to visualize in Gephi (v 0.9.2). Edge widths were scaled to represent Spearman correlation size, undirected network diameters for node sizes were calculated using a betweenness centrality parameter that emphasizes nodes that are central to the network. Nodes were colored by module group calculated by the “cluster_louvain” algorithm, a community assignment for vertices (nodes or groups of nodes) that maximizes contribution to modularity (Csardi and Nepusz, 2006; Blondel et al., 2008). Modules were then queried through FUNGuild (Nguyen et al., 2016) and FungalTraits (Põlme et al., 2020) to provide functional characteristics for each module, split by the weighted relative abundance of each ASV.

## Results

### Microscopy and Culturing

**Figure 3:**
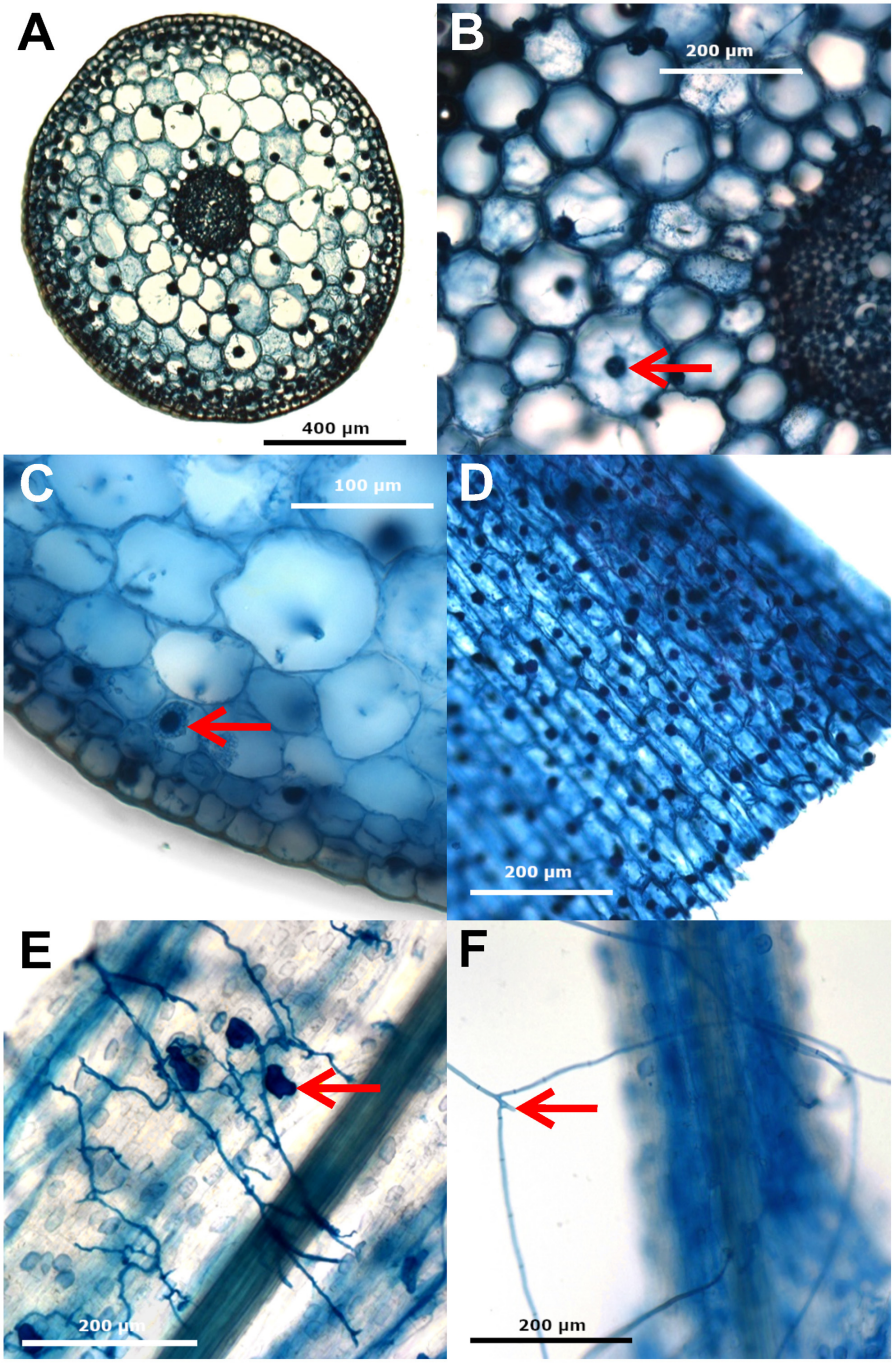
Images of *Cypripedium reginae* roots (AO-Orch) **(A)** in transverse section, **(B,C)** transverse section with likely condensed/collapsed pelotons as dark circles within root cells (arrows), **(D)** in longitudinal section, and *Fraxinus nigra* roots (AO-Ash), **(E)** in transverse section showing branched hyphae and likely vesicles (arrow), and **(F)** simple septate hyphae (arrow) externally associated with root tips. Roots were stained with trypan blue and images taken using a ZeissZ1 microscope with brightfield illumination.

Sections of both orchid and black ash roots showed non-mycorrhizal associations and probable orchid mycorrhizal and arbuscular mycorrhizal structures (Figure 3). Within orchid samples, several examples of collapsed pelotons are visible along with some thin intra- and intercellular hyphae, which are typical of orchid mycorrhizal associations (Figures 3A–D). The black ash sections show possible arbuscular mycorrhizal (Glomeromycota) vesicles and hyphae (Figure 3E) and external hyphae of root-associated fungi (Figure 3F). Most sections showing putative AMF structures were from black ash roots, whereas orchid roots primarily had collapsed peloton structures, with other intra- and intercellular hyphae present less often. No mantle or Hartig net, characteristic of ectomycorrhizae were observed in either root type, nor were clamp connections, diagnostic of Basidiomycota, present on any mounts. No growth of fungi or bacteria was recorded on MEA plates from any of the SS or NSS root tips 2 weeks after stabbing them into the agar prior to DNA extraction; the time in CTAB had rendered all root-associated microbes non-viable, whether surface-sterilized or not (data not shown).

### Sequencing Output

After manual filtration and removal of non-target ASVs, there were 993 ASVs with a total of 630,513 sequences across 51 samples (not including controls or three SS orchid samples with <1,000 read depth) that were used for downstream analyses (Supplementary Table 2). Surface sterilized orchid root samples had lower average read depth (an average of 1,073 sequences/sample) than ash roots (average 22,764 sequences/sample). Sample reads were not rarified since there was biological relevance to the nearly 10-fold difference in sequencing depths of orchid and ash.

### Alpha Diversity

**Figure 4:**
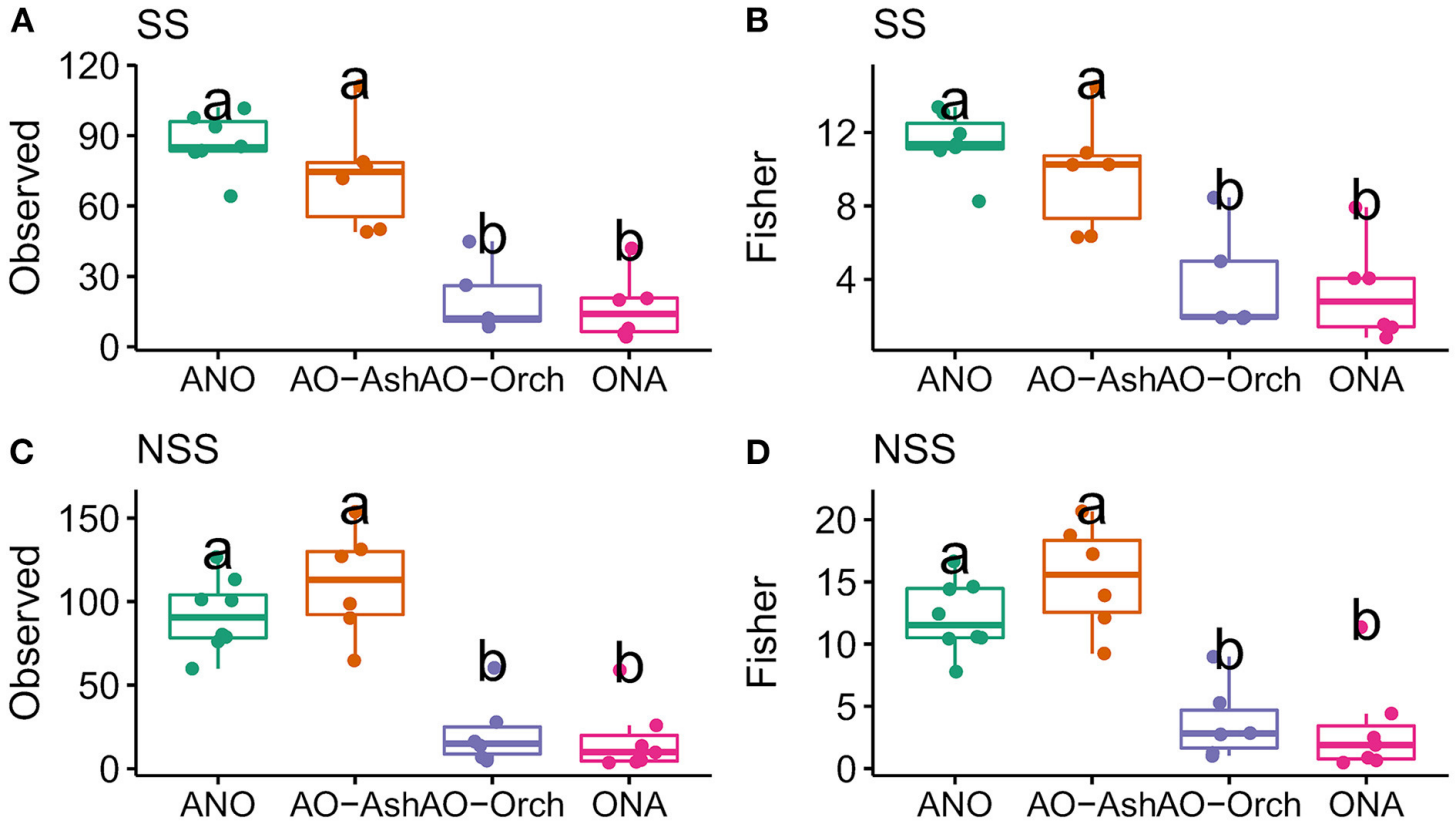
Alpha diversity metrics for surface-sterilized and non-surface-sterilized black ash (*Fraxinus nigra*) and showy lady's slipper (*Cypripedium reginae*) roots: ITS2 **(A)** Observed richness, **(B)** Fisher's alpha diversity index of surface-sterilized roots, **(C)** Observed richness, and **(D)** Fisher's alpha diversity index of non-surface-sterilized roots. Orchid and ash sample groups: ANO, ash, no orchid; AO-Ash, ash near orchid; AO-Orch, orchid near ash; ONA, orchid, no ash. The same letters within each plot identify samples that are not significantly different from one another.

Alpha diversities were measured for orchid and ash samples split by SS and NSS roots. Ash roots had greater ASV richness (observed) and diversity (Shannon and Fisher's alpha) than orchid roots in both SS and NSS samples (Figure 4; Supplementary Table 3). Relative proportions of fungi at the order level differed between sample groups after surface-sterilization (SS). Glomerales dominated ash roots after surface-sterilization, particularly in ash with orchids nearby (Supplementary Figure 1). In non-surface-sterilized roots (NSS), most sequences (62.4%) but only 112 ASVs were found in both orchid and ash roots, whereas 37.3% of the sequences and many more (625) ASVs were found only in ash roots, and 0.6% of sequences and 37 ASVs were found only in orchid roots (Supplementary Figure 2A). After surface-sterilization, there was a 30% (232 ASV) reduction of fungal sequences, with the majority (51.2% of sequences and 449 ASVs) found only in SS ash roots, 0.7% of sequences and 21 ASVs found only in SS orchid roots, and 48.1% and 66 ASVs found in both orchid and ash roots (Supplementary Figure 2B).

### Beta Diversity

Mean ASV comparison between groups showed no significant difference between SS and NSS samples (*p* = 0.48) or sample processing batch effects (*p* = 0.31), but there were significant differences between orchid and ash treatment groups (*p* = 1 × 10^−4^, ANOSIM R = 0.5153) and sampling location (*p* = 9 × 10^−4^, ANOSIM R = 0.1997). Comparing sample (Bray-Curtis) distances within treatment groups using a Kruskal-Wallis test yielded no significant differences between treatment groups (*p* = 0.9), but there were significant differences by location (*p* = 3.7 × 10^−4^) (Supplementary Figure 3). Location is likely linked with both orchid and ash treatment groups and ASV community structure since sometimes only one treatment type was found in a location (e.g., Grenfell Campus fen and Corner Brook fen only had ONA samples), and there were noticeable site-specific differences as mentioned in the Sampling section.

**Figure 5:**
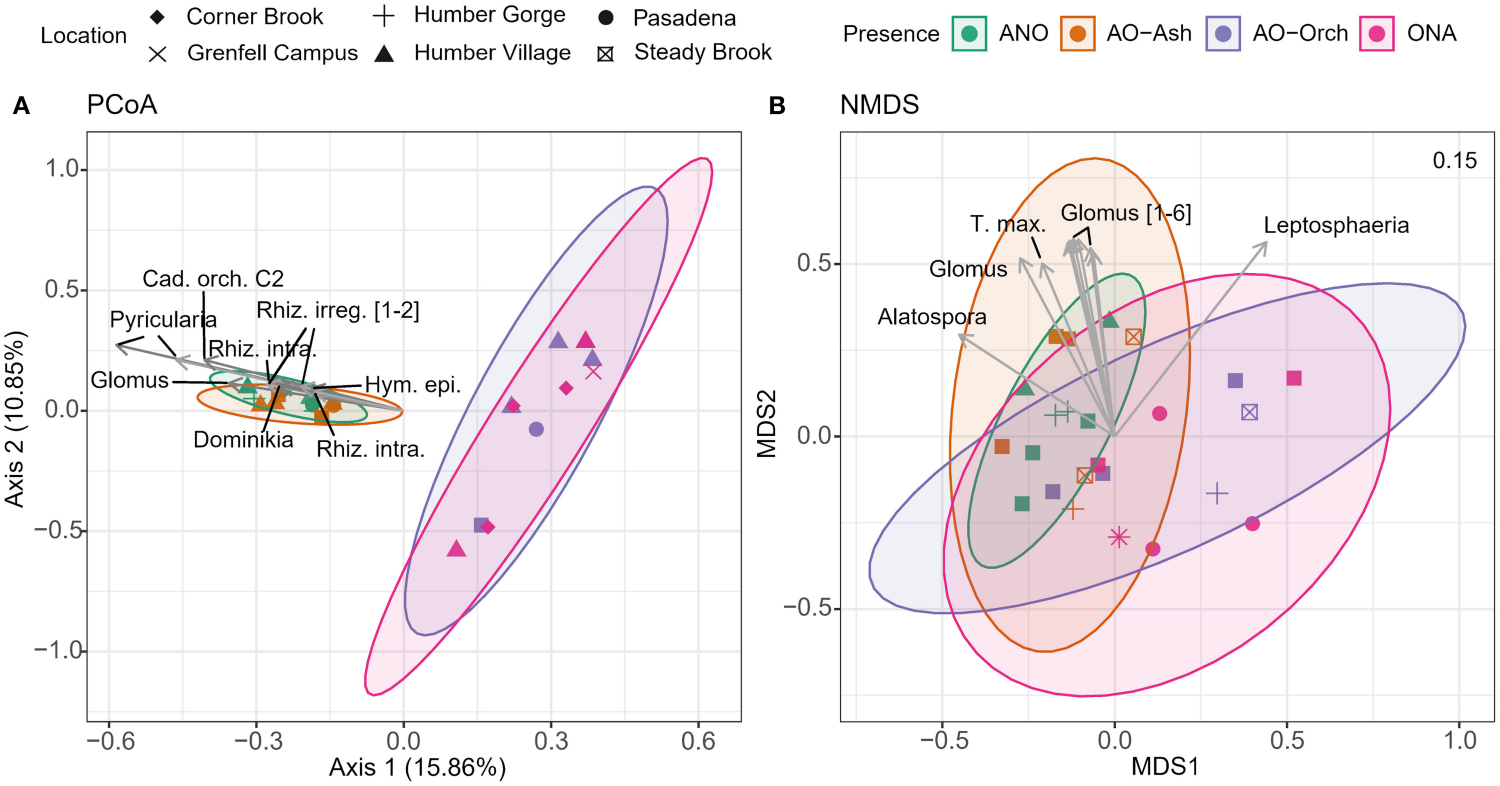
**(A)** PCoA and **(B)** NMDS of ITS2 ASVs recovered from surface-sterilized roots. All samples are identified by orchid and ash sample groups (ANO, ash, no orchid; AO-Ash, ash near orchid; AO-Orch, orchid near ash; ONA, orchid, no ash) and location. PCoA sample distances calculated using Bray-Curtis. NMDS model stress values are included. Abbreviated ASVs include: Cad. orch., *Cadophora orchidicola*; Hym. epi., *Hymenoscyphus epiphyllus*; Rhiz. intra., *Rhizophagus intraradices*; Rhiz. irreg., *Rhizophagus irregularis*; T. max., *Tetracladium maxilliforme*. Where present, values in square brackets after each label indicate the number of clustered vectors [e.g., Glomus (1–6) means there are six *Glomus* sp. vectors].

A PCoA (based on linear mapping) shows greater within sample diversity in both orchid root treatments (ONA and AO-Orch) than roots of black ash (Figure 5). In comparison to ash roots that had multiple fungal associates forming more similar communities, roots of orchids were dominated by quite different fungi (Supplementary Figure 4).

Surface-sterilized ash roots have a substantially higher read depth than orchids (all of the top 10 correlated ASVs were specific to ash samples) (Figure 5). Notable taxa in ash included the broad-host root endophyte *Cadophora orchidicola* (Fernando and Currah, 1996), *Pyricularia*, and *Hymenoscyphus epiphyllus*, and the AMF *Glomus, Rhizophagus irregularis, R. intraradices*, and *Dominikia* (Glomeraceae). Ordination in NMDS (based on rank distribution and non-linear mapping) revealed that community proportions between samples are more similar, with slight variations due to location and orchid/ash presence. Relative to sample differentiation, there were 10 significant (*p* < 0.05) ASVs, of which seven are *Glomus* in ash roots, plus *Tetracladium maxilliforme, Leptosphaeria*, and *Alatospora*.

Non-surface-sterilized roots of ash had substantially more *Dactylonectria macrodidyma* and *Xylomyces aquaticus* than orchids (PCoA) (Supplementary Figure 4A) and were significantly (*p* < 0.05) dominated by AMF (*Glomus* spp., *Dominikia*); other significant taxa without any obvious location or presence-based influences included *Amphinema* sp., *Hymenoscyphus epiphyllus, Ilyonectria radicicola*, and *Thelephora alnii* (NMDS) (Supplementary Figure 4B).

### Shared Fungi Between Orchid and Ash

**Figure 6:**
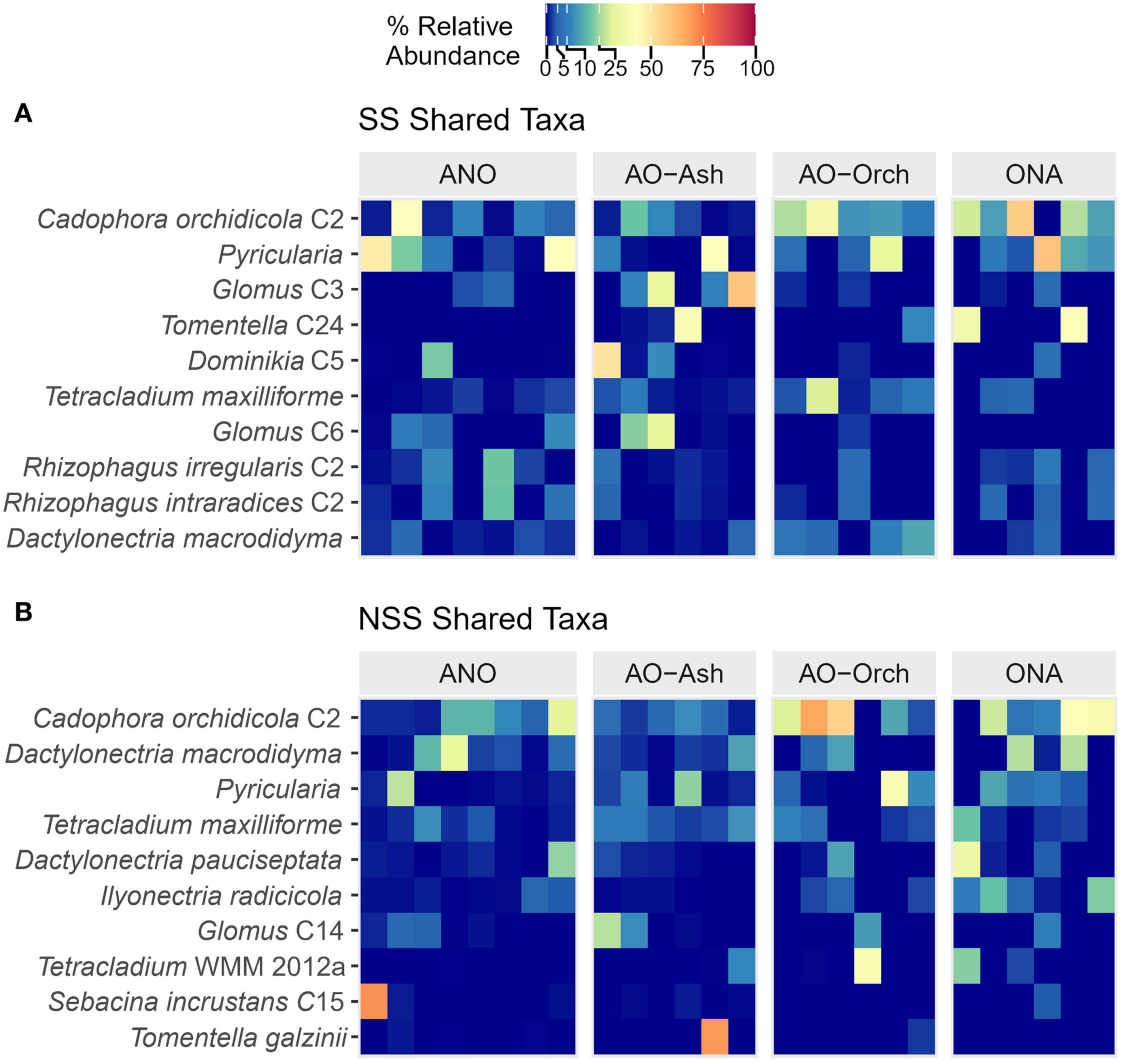
Heatmap of the top 10 abundant fungal genera (ITS2) found between shared **(A)** surface-sterilized (SS) and **(B)** non-surface-sterilized (NSS) roots of black ash (*Fraxinus nigra*) and showy lady's slipper orchid (*Cypripedium reginae*). Sample groups: ANO, ash, no orchid; AO-Ash, ash near orchid; AO-Orch, orchid near ash; ONA, orchid, no ash. Identical names followed by the same clade (C) number signify ASVs that are within the same clade in maximum likelihood analysis, with <0.01 evolutionary distance, and likely belong to a single organism.

There were 112 ASVs shared between NSS roots of orchid and ash and 66 ASVs shared between SS roots (Supplementary Figure 2); all 66 shared SS ASVs were also present in NSS shared samples (Supplementary Table 4). The top ASV clade (by relative abundance) shared between both SS and NSS orchid and ash roots was *Cadophora orchidicola* C2 (Figure 6). Root pathogens *Dactylonectria macrodidyma, D. pauciseptata, Ilyonectria radicicola* and *Pyricularia*, and saprotroph *Tetracladium maxilliforme* are also present in high abundance in both SS and NSS roots. Twenty-six of the 66 shared ASVs are arbuscular mycorrhizal *Glomus, G. macrocarpum, Rhizophagus intraradices, R. irregularis*, with lower read numbers of *Claroideoglomus claroideum* and *Dominikia* (Supplementary Table 4). Roots of SS had higher relative abundances of AMF, whereas NSS roots had more root pathogens. Several ectomycorrhizal fungi were also present in SS roots of both *Cypripedium* and *Fraxinus*, including two *Tomentella* sp. clades (C4 and C24), one *T. galzinii* C4, and *Sebacina incrustans* C5. Both SS and NSS orchid root samples had single, dominating ASVs or clades (*Tomentella* C4, *Hyaloscypha finlandica, Tomentella* C9, *Ceratobasidium, Dominikia* C1, *Leptosphaeria*) nearing 75–100% relative abundance in single samples—i.e., orchid roots had more differential, unique taxa. In contrast, ash roots had single ASVs or clades with more equal abundances across samples, without single dominants (Supplementary Figure 5).

### Network Analysis

**Figure 7:**
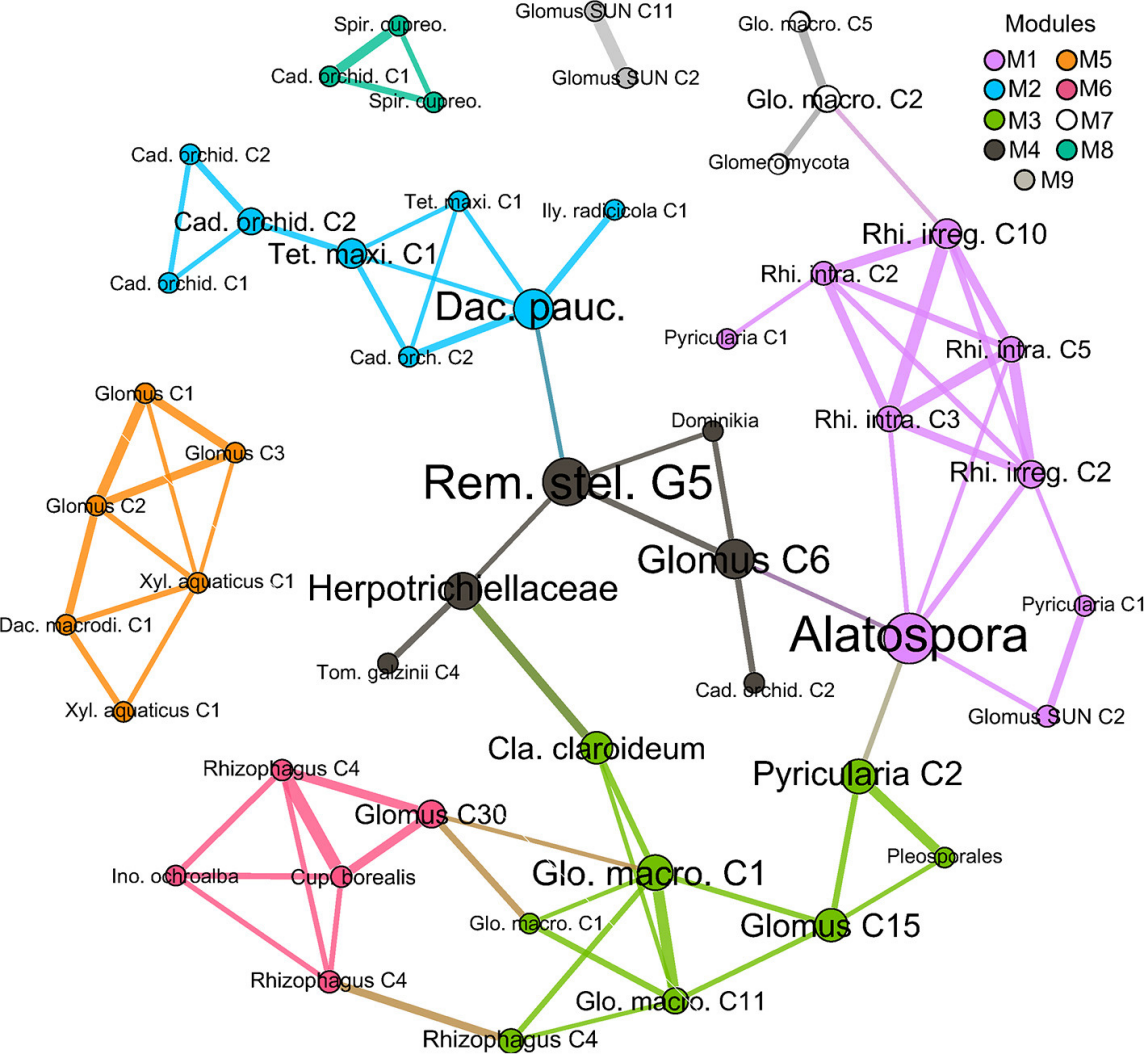
Correlation network of fungi occurring in surface-sterilized roots of both black ash (*Fraxinus nigra*) and showy lady's slipper orchid (*Cypripedium reginae*), based on ITS2 sequence data (ASVs grouped as clades <0.01 distance in ML phylogeny). Edges between nodes represent a strong positive Spearman's correlation (ρ > +0.57). Modules are grouped by color, edge widths are scaled by correlation size, and node sizes are proportional to the number of connections. Duplicate ASV clades sharing the same genus are numbered. Shortened taxon names include: Cad. orchid., *Cadophora orchidicola*; Cla. claroideum, *Claroideoglomus claroideum*; Cup. borealis, *Cuphophyllus borealis*; Dac. pauc., *Dactylonectria paucispora*; Ily. radicicola, *Ilyonectria radicicola*; Ino. ochroalba, *Inocybe ochroalba*; Glo. macro., *Glomus macrocarpum*; Rem. stel., *Remispora stellata*; Rhi. irreg., *Rhizophagus irregularis*; Rhi. intra., *Rhizophagus intraradices*; Spir. cupreo., *Spirosphaera cupreorufescens*; Tet. maxi., *Tetracladium maxilliforme*; Tom. galzinii, *Tomentella galzinii*; Xyl. aquaticus, *Xylomyces aquaticus*.

A network analysis was performed using all 66 shared SS ASVs, labeled with 38 distinct taxonomic clades (evolutionary distance <0.01) in a ML tree (Figure 7). Overall, the network of fungi found to be shared by orchid and ash roots was composed of nine separate modules, with several peripheral nodes that interconnected six modules, and three entirely disconnected modules. All network members were positively correlated (ρ ≥ + 0.57). The most common nodes were those of arbuscular mycorrhizal clades (*Glomus* and *Rhizophagus*), followed by *Cadophora orchidicola*, and a plant pathogen (*Pyricularia*). Peripheral nodes with high among-module connectivity (*Pi* > 0.4) and high within-module connectivity (*Zi* > 0) included *Glomus* C6, *Alatospora, Rhizophagus* C4, *R. irregularis* C10, *Glomus* C15, and *T. maxilliforme* C1 (Supplementary Figure 6). Highly correlated clusters of *C. orchidicola* (module 2), *Glomus* sp. 3 SUN 2011 G2 (module 9), *Spirosphaera cupreorufescens* G1 (module 8) suggest the presence of artifactual or genetic variants of the same organism. Neither FungalTraits nor FUNGuild had matches for *Dominikia*, and *Cadophora orchidicola* ASVs had to be searched under its synonym *Leptodontidium orchidicola* in FungalTraits, with FUNGuild lacking matches to either synonym, but instead matching for Helotiaceae (Supplementary Figure 7).

## Discussion

Mycoheterotrophic orchid species often show high specificity to a narrow range of fungi that are also capable of forming mycorrhizae with surrounding plants (Bidartondo and Bruns, 2001; Julou et al., 2005). Here we present evidence of multiple shared mycorrhizal fungi—ericoid (*Cadophora orchidicola*), arbuscular (*Claroideoglomus claroideum, Dominikia, Glomus, Rhizophagus irregularis* and *R. intraradices*), and ectomycorrhizal (*Inocybe, Sebacina incrustans*, and *Tomentella*)—between the showy lady's slipper orchid (*Cypripedium reginae*) and black ash (*Fraxinus nigra*) growing in a temperate-boreal region. Prior to this study, the only fungal associate found in *C. reginae* was *Rhizoctonia sclerotica* (Ceratobasidiaceae) (Curtis, 1939). The largest proportion of ITS2 fungal reads belonged to Ascomycota (394 ASVs, 42.3%), followed by Glomeromycota (383 ASVs, 39.9%), and then Basidiomycota (212 ASVs, 17.8%). Surface-sterilization resulted in the loss of 232 ASVs from all samples, with ash losing a larger proportion than orchids. Non-surface-sterilized samples of orchids and ash within 15 m of one another (AO-Orch and AO-Ash) had 152 co-occurring ASVs; after surface-sterilization, there were 66 shared ASVs belonging to 44 clades (ML evolutionary distance <0.1) between orchid and ash. This loss of ASV richness is a better measure of the proportion of fungi restricted to the root surfaces than was the attempt to culture fungi and bacteria from SS and NSS root tips. Due to the nearly 2,000 km distance between sampling and lab, the samples we plated and extracted DNAs from were preserved in CTAB (Gardes and Bruns, 1993), which in addition to protecting the DNA from degradation also disrupted cell membranes resulting in loss of cell viability. Nonetheless, since the bleach step of the surface-sterilization protocol likely degraded the DNA of fungi restricted to the surface of roots, this protocol served the purpose it was intended for, which was to better distinguish truly endophytic fungi.

Twenty-six ASVs (of 66) of AMF were shared between orchid and ash, including *Claroideoglomus claroideum, Dominikia*, seven *Glomus*, five *G. macrocarpum*, three *Glomus* sp. 2 SUN 2011, three *Rhizophagus*, three *R. intraradices*, two *R. irregularis*, and an unknown Glomeromycota. Other studies have identified *Glomus* and *Rhizophagus clarus* (as *Glomus clarum*) in *Cypripedium californicum*, and *Funneliformis mosseae* (as *Glomus mosseae*) in *C. parviflorum* (Shefferson et al., 2005), as well as *Glomus, Gigaspora, Scutellospora, Claroideoglomus* and other likely AMF in other Orchidaceae (Gebauer and Meyer, 2003; Bidartondo et al., 2004; Voyron et al., 2017; Voronina et al., 2018). Notably, no AMF were unique to SS ash roots. Instead, all Glomeromycetes found in SS ash roots were also found in SS orchid roots (Supplementary Table 4).

Fewer ectomycorrhizal fungi were seen (five of 66 ASVs), including *Sebacina incrustans*, a member of a group that, in addition to forming ectomycorrhizae with a broad range of host plants, may also form mycorrhizae in orchids (Urban et al., 2003), and *Inocybe ochroalba* (Kuyper, 1986; Peintner and Horak, 2002; Ryberg et al., 2008), *Tomentella* spp. and *T. galzinii* (Selosse et al., 2006), obligate ectomycorrhizal basidiomycetes that have been found in Orchidaceae roots (Bidartondo et al., 2004; Xing et al., 2020; Suetsugu et al., 2021). The low number of ECM shared between *C. reginae* and *F. nigra* may likely be because of the influence of ash, since it is preferentially colonized by AMF (Malloch and Malloch, 1982; Brundrett et al., 1990), and may facilitate the establishment of understory species that are also AMF-associating (Veresoglou et al., 2017).

Shefferson et al. (2005) identified the primary mycorrhizal symbionts in 59 plants of seven *Cypripedium* species (not including *C. reginae*) across the northern hemisphere. Fungi from the Tulasnellaceae dominated mycorrhizal tissue across all species; other fungal groups included *Phialophora*, Sebacinaceae, Ceratobasidiaceae, Thelephoraceae, *Glomus, Russula*, and Agaricales, in order from most to fewest number of plants infected. We chose to use the primers 5.8S-Fun and ITS4-Fun, which were recommended for their amplification of a broad range of Fungi (Taylor et al., 2016). However, future studies of this system should pair these primers with the 5.8S-Tulngs and ITS4-Tul2 primer pair ((Rammitsu et al., 2021)), since the primers used in this study have a strong bias against members of the Tulasnellaceae. In addition, future studies should also sample orchid roots further from their tips, where there may be greater mycorrhizal colonization (Calevo et al., 2021).

There were no obvious sampling location trends in PCoA and NMDS beta diversity analyses of SS roots, and confidence ellipses for each sample type were highly overlapping in NMDS (Figure 5). This suggests that ASV communities in these locations that are shared between hosts are relatively similar. Beta diversity analyses of the 66 shared SS orchid and ash fungi highlighted similar key symbionts in this system, including at least three clades of *Cadophora orchidicola* [=*Leptodontidium orchidicola*], an ericoid mycorrhizal fungus and litter saprotroph (Rasmussen, 1995; Fernando and Currah, 1996), which had the highest relative abundance in both SS and NSS roots of *Cypripedium* and *Fraxinus* (Figure 6). In axenic synthesis experiments with different plant hosts, *L. orchidicola* formed associations ranging from mutualistic and mycorrhizal through to one-sided and pathogenic, including invasion of the stele in *Salix*, causing extensive cellular lysis (Fernando and Currah, 1996). It could be that *Cypripedium* is able to control root invasion by *L. orchidicola* to its advantage, as is the case in multiple other orchid mycorrhizal associations with otherwise phytopathogenic fungi, from *Armillaria* to *Rhizoctonia* (Burgeff, 1959).

Approximately one third (20 of 66 clades) of shared endophytic fungi were litter, soil, or wood saprotrophs, and one sixth (11 of 66 clades) were plant pathogens (Supplementary Table 4). Non-surface-sterilized ash, and surface-sterilized orchids and ash had high proportions of phytopathogens, including *Xylomyces aquaticus* [=*Vargamyces aquaticus*] (Gonczol et al., 1990; Pinnoi et al., 2006), *Dactylonectria macrodidyma* [=*Cylindrocarpon macrodidyma*] (Halleen et al., 2004), *Ilyonectria radicicola* (Cabral et al., 2012) and *Pyricularia* (Hyde et al., 2020), suggesting that all were in high abundance, within and at the root surface, of both plants. The relationship of endophytic fungi may vary from more harmful to more harmonious, depending on the orchid host. *Armillaria* is a facultative necrotrophic fungus that typically colonizes and kills living root tissue to obtain nutrients, however, it also colonizes the achlorophyllous orchids *Galeola* and *Gastrodia* without exhibiting foliar or root symptoms (Kikuchi et al., 2008), so it is likely acting as a nutrient host to the orchids (Baumgartner et al., 2011). Earlier observations of *Gastrodia minor* also show root collapse after an unknown fungal infection (Campbell, 1963), inferring a pathogenic interaction. Sections of *C. reginae* did not show any root collapse suggesting a fungal infection, so pathogen pressure may be on ash and other plants acting as nutrient sources in this system (Baumgartner et al., 2011). Despite different ecological roles, both pathogens and saprotrophs have been shown to support mycoheterotrophy (Campbell, 1970; Bidartondo et al., 2002; Kikuchi et al., 2008; Baumgartner et al., 2011).

*Cuphophyllus borealis*, a symbiotroph-saprotroph waxcap, occurred in only four samples—twice in NSS ash, and once each in SS ash and orchid across multiple locations—and may be an interesting root endophyte in the *C. reginae* and *F. nigra* system in this region. A preliminary metabarcoding analysis of a single sample from the Grenfell Campus fen identified an OTU as *Cuphophyllus virgineus* (Chatzidakis, 2013), which is likely the same taxon (Lodge et al., 2014). The single sequence appears as the only basidiomycete peripheral node within the SS correlation network, with a high among-module and within-module connectivity scores. Within the network, *C. borealis* was correlated with multiple mycorrhizal fungi: two AMF *Rhizophagus* C4 ASVs, a *Glomus* C30 clade, and ECM *Inocybe ochroalba* in module 5 which also connects to another AMF-dominated module 3. *Inocybe ochroalba* has been sequenced from the roots of Eurasian chlorophyllous orchids *Gymnadenia conopsea* and *Epipactis helleborine* (Xing et al., 2020), of which the latter is now common as an invasive species in Newfoundland (Voitk and Voitk, 2006). In addition to detecting *I. ochroalba* in SS roots of both orchid and ash, we also found it on NSS orchid roots, together with other *Inocybe* species (*I. geophylla, I. griseolilacina*, and *I. sindonia*).

Non-surface-sterilized samples of ash had high proportions of plant pathogenic fungi, including *Xylomyces aquaticus* [=*Vargamyces aquaticus*] (Gonczol et al., 1990; Pinnoi et al., 2006), *Dactylonectria macrodidyma* [=*Cylindrocarpon macrodidyma*] (Halleen et al., 2004), *Ilyonectria radicicola* (Cabral et al., 2012), and *Pyricularia* (Hyde et al., 2020), suggesting that all were in high abundance at the root surface of both plants. These and other fungal pathogens of plant roots are another possible avenue of nutrient transfer from ash to orchid, since all are found within ash and orchid roots when growing nearby, as well as in high abundance on the surface of ash roots.

As in the *C. reginae* in this study, *Cephalanthera damasonium*, investigated by Julou et al. (2005), had multiple groups of fungal partners; many ECM fungi in the Basidiomycota (Thelephoraceae and Cortinariaceae) or Ascomycota (Pezizales); ascomycetous root biotrophs and mycorrhizal species (Helotiales) and ‘dark septate root endophytes' (*Cadophora orchidicola, Phialophora* and *Exophiala* spp.; Rasmussen, 1995; Jumpponen and Trappe, 1998); and a third group of ascomycetous plant parasites (Nectriaceae spp.) and saprobes (Sordariales). No AMF (Glomales) were found in association with *C. damasonium*, likely because it was found growing in a closed canopy alongside *Quercus robur* and *Corylus avellana* forest with other herbaceous plants and shrubs, surrounded by photosynthetic plants that form ECM associations. The AMF partners that we detected in *C. reginae* roots may be driven by the presence of the black ash nearby, a known AMF host (Malloch and Malloch, 1982; Brundrett et al., 1990). This possible relationship should be further investigated by tracking the carbon and nutrient transfer between orchid and ash.

## Conclusion

We have demonstrated the presence of a diversity of fungi, including taxa that are known to form ericoid, arbuscular, and ectomycorrhizal associations as well as some that are pathogens or are of unknown nutritional modes, in the roots of both adult chlorophyllous lady's slipper orchids and black ash in Newfoundland fens. This raises the possibility that one or more of these shared fungi could be conveying carbon and nutrients between these two plants, and possibly others as well, in a manner similar to that of common mycorrhizal networks connecting other plant species (Simard and Durall, 2004; Selosse et al., 2006). Tracer studies are required to demonstrate any such carbon and nutrient flow and its magnitude and direction and could finally provide answers to the enigmatic question of why showy lady's slippers are so frequently found surrounding black ash in this boreal environment. Since the orchid roots hosted fungi with such a broad range of other potential connections—with ericoid shrubs, arbuscular mycorrhizal graminoids, broad-leaved herbs and trees, and ectomycorrhizal Betulaceae, Pinaceae and Salicaceae—a much broader metabarcoding study of the fungi associated with identified, surface-sterilized roots of many other species in the plant community could turn up an unrecognized multispecies nutritional network below ground. Again, tracer studies would be required to document the strength and directions of the linkages, and the importance of particular species as network nodes.

## Data Availability Statement

The datasets presented in this study can be found in online repositories. The names of the repository/repositories and accession number(s) can be found at: https://www.ebi.ac.uk/ena, PRJEB47630.

## Author Contributions

RT and DS conceived and designed the analysis. KK and RS contributed to sample analysis and wrote the initial draft. NW performed statistical analyses, prepared figures, and compiled the results into the final manuscript, in consultation with RT. All authors contributed to and approved the final manuscript.

## Funding

Funding for work by KK, RS, and RT was provided by the Faculty of Science and Department of Biology, University of Western Ontario; for NW from a generous donation to the Dr. Laurie L. Consaul Herbarium (UWO); for DS by Grenfell Campus, Memorial University of Newfoundland and Labrador.

## Conflict of Interest

The authors declare that the research was conducted in the absence of any commercial or financial relationships that could be construed as a potential conflict of interest.

## Publisher's Note

All claims expressed in this article are solely those of the authors and do not necessarily represent those of their affiliated organizations, or those of the publisher, the editors and the reviewers. Any product that may be evaluated in this article, or claim that may be made by its manufacturer, is not guaranteed or endorsed by the publisher.
