# Correction: Multiple fungi may connect the roots of an orchid (*Cypripedium reginae*) and ash (*Fraxinus nigra*) in western Newfoundland

**DOI:** 10.3389/ffunb.2025.1691090

**Published:** 2025-10-08

**Authors:** Nimalka M. Weerasuriya, Katarina Kukolj, Rebecca Spencer, Dmitry Sveshnikov, R. Greg Thorn

**Affiliations:** ^1^ Department of Biology, University of Western Ontario, London, ON, Canada; ^2^ School of Science and the Environment, Grenfell Campus, Memorial University, Corner Brook, NL, Canada

**Keywords:** showy lady’s slipper orchid, black ash, mycorrhiza, metabarcoding, Illumina MiSeq, mixotrophy, common mycorrhizal network, ITS2

There was a mistake in **Supplementary Data Sheet 1** as published. **Supplementary Figure 1** was composed incorrectly, with different colours representing the same taxa in left and right panels. Minor typographical corrections were also made to the caption of **Supplementary Figure 3**.

The original version of this article has been updated.

