# Supplementary material for: Multiple Fungi May Connect the Roots of an Orchid (Cypripedium reginae) and Ash (Fraxinus nigra) in Western Newfoundland
Source: Front Fungal Biol. 2022 Mar 1;3:805127. doi: 10.3389/ffunb.2022.805127 (PMC10512338; doi:10.3389/ffunb.2022.805127)
Supplement: Supplementary file 1 [file Data_Sheet_1.pdf]

Supplementary Material

1 Supplementary Data

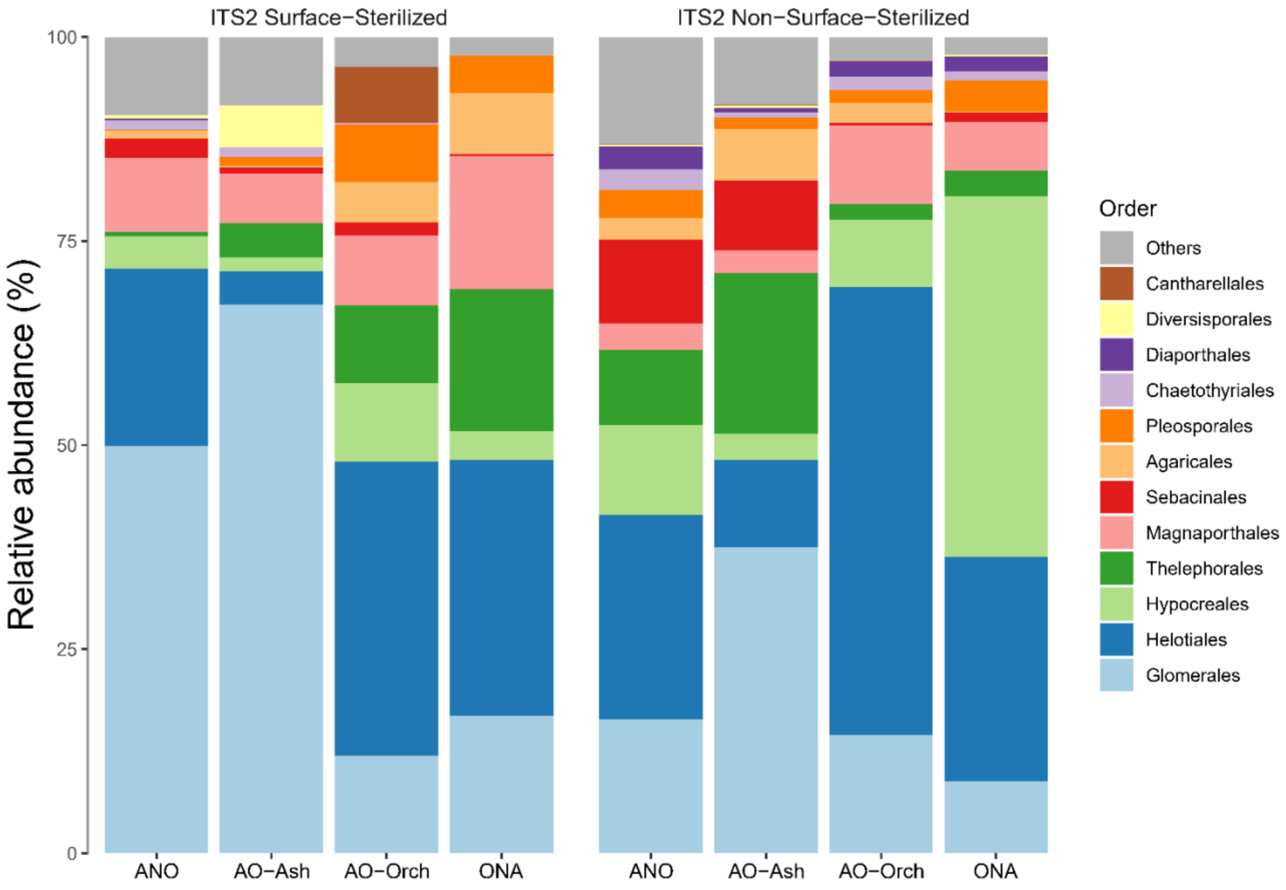

Supplementary Figure 1. Stacked relative abundances of (left) ITS2 surface-sterilized, and (right) ITS2 non-surface-sterilized ASVs found in showy lady's slipper orchid (*Cypripedium reginae*) and black ash (*Fraxinus nigra*) roots. Sample groups: ANO – ash, no orchid; AO-Ash – ash near orchid, AO-Orch – orchid near ash, and ONA – orchid, no ash.

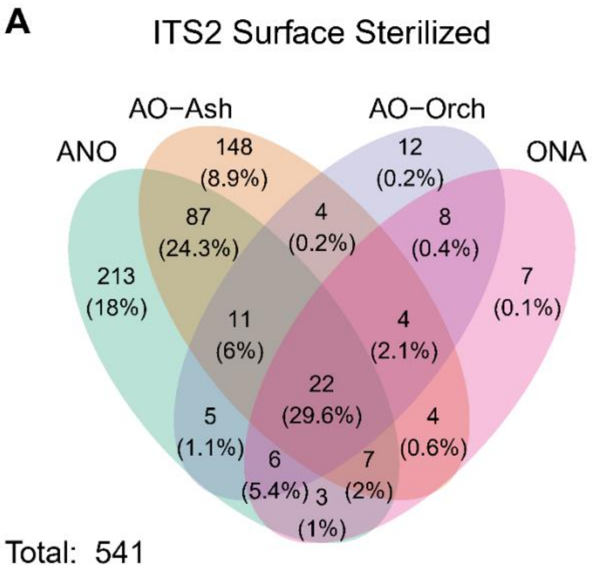

Supplementary Figure 2. A Venn diagram of ITS (A) surface-sterilized, and (B) non-surface-sterilized roots of showy lady's slipper orchid (*Cypripedium reginae*) and black ash (*Fraxinus nigra*) and fungal taxa. Sample groups: ANO – ash, no orchid; AO-Ash – ash near orchid, AO-Orch – orchid near ash, and ONA – orchid, no ash. Counts are distinct (unclustered) ASVs and percentages are the proportion of read counts from the total.

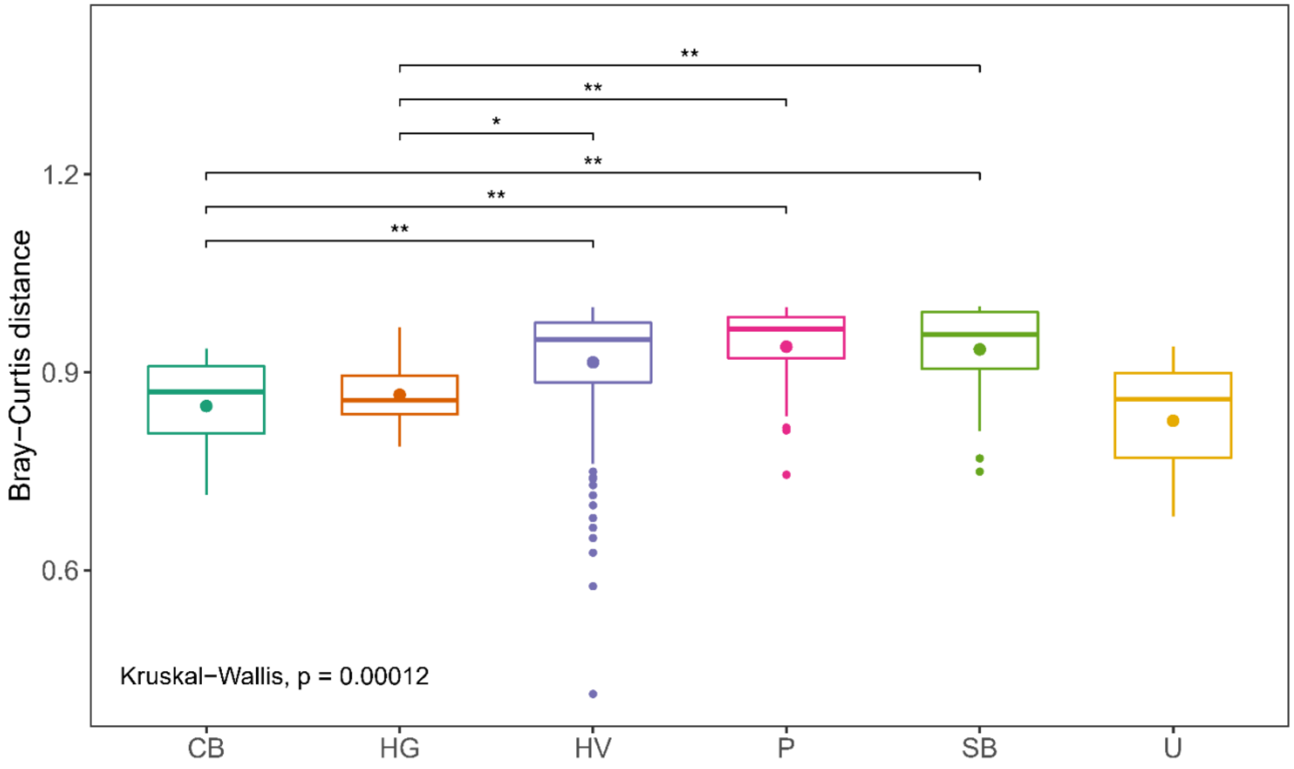

Supplementary Figure 3. A comparison of ITS2 ASV counts, from showy lady's slipper orchid (*Cypripedium reginae*) and black ash (*Fraxinus nigra*) roots, separated by location. Location codes: CB – Corner Brook, HG – Humber Gorge, HV – Humber Village, P – Pasadena, SB – Steady Brook, U – Grenfell Campus, Memorial University. Dots within each boxplot represent group means. Significance codes: * as p-value < 0.05, ** as p-value < 0.01, *** as p-value < 0.001, and *** as p-value < 0.0001. P-values based on Dunn's test of multiple comparisons after Benjamini-Hochberg p-value adjustment.

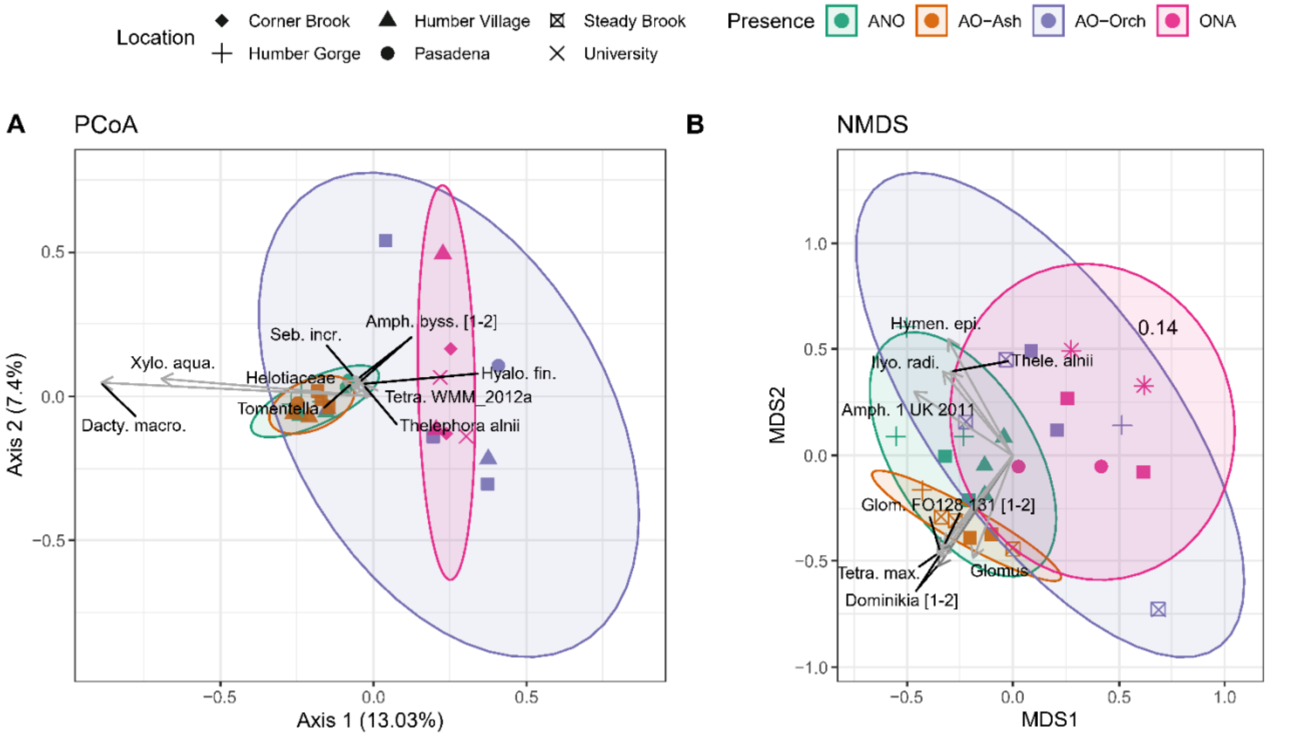

Supplementary Figure 4. (A) PCoA and (B) NMDS of non-surface-sterilized of showy lady's slipper orchid (*Cypripedium reginae*) and black ash (*Fraxinus nigra*) roots. All samples are identified by orchid-ash presence (ANO – ash, no orchid; AO-Ash – ash near orchid, AO-Orch – orchid near ash, and ONA – orchid, no ash) and location. PCoA sample distances calculated using Bray-Curtis. NMDS model stress values are included. Abbreviated ITS2 ASVs include: Amph. byss. – *Amphinema byssoides*, Dacty. macro. – *Dactylonectria macrodidyma*, Glom. – *Glomus*, Hyalo. fin. – *Hyaloscypha finlandica*, Hymen. epi. – *Hymenoscyphus epiphyllus*, Ilyo. radi. – *Ilyonectria radicicola*, Seb. incr. – *Sebacina incrustans*. Tetra. max. – *Tetracladium maxilliforme*, Thele. alnii – *Thelephora alnii*, Xylo. aqua. – *Xylomyces aquaticus*. Where present, values in square brackets after each label indicate the number of clustered vectors (e.g., *Dominika* [1-2] means there are two *Dominika* vectors).

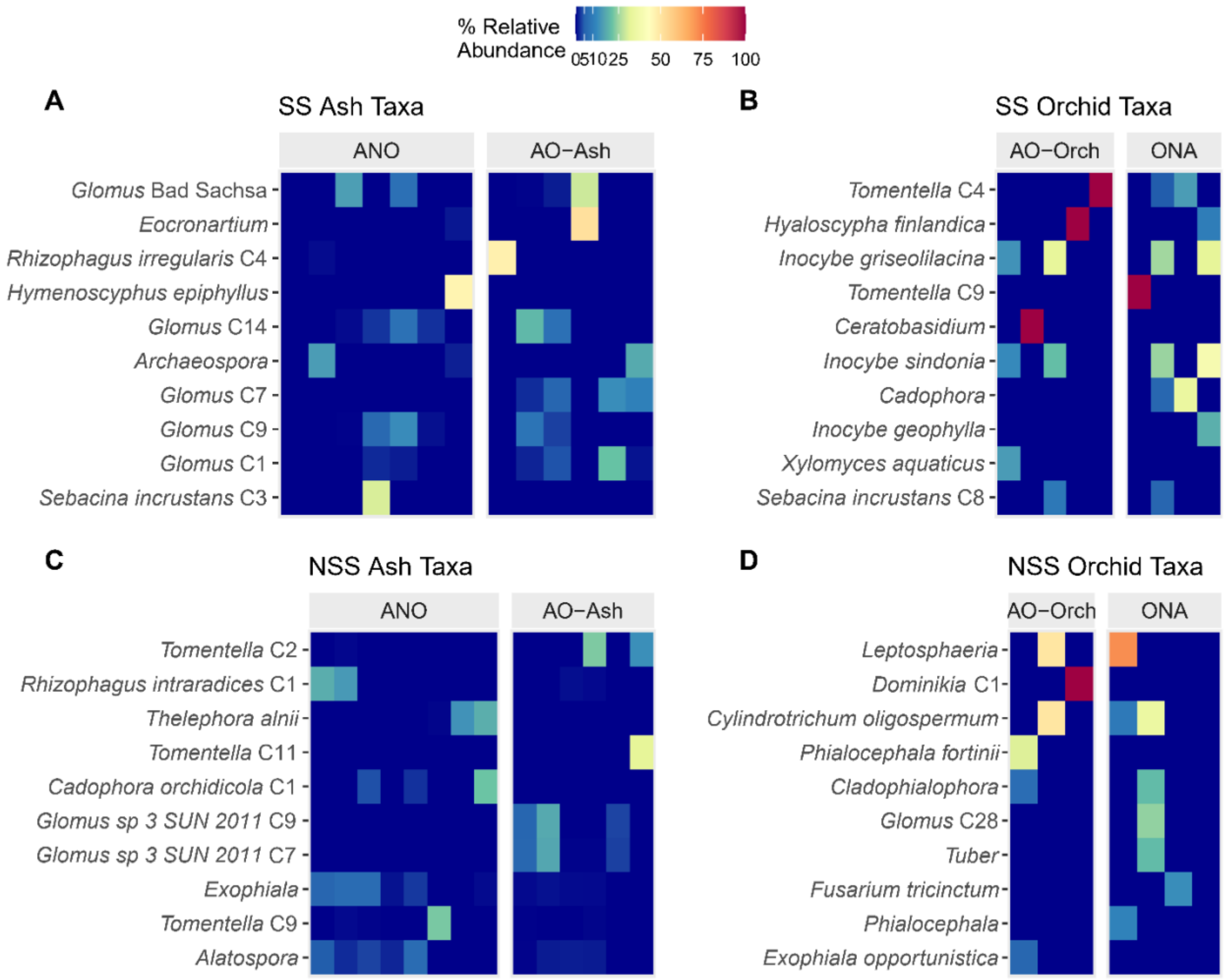

Supplementary Figure 5. Heatmap of the top 10 abundant fungal genera (ITS2) found unique to black ash (*Fraxinus nigra*) and showy lady's slipper orchid (*Cypripedium reginae*). Panels split samples by (A) surface-sterilized (SS) ash, (B) SS orchid, (B) non-surface-sterilized (NSS) ash, and (C) NSS orchid. Sample groups: ANO – ash, no orchid; AO-Ash – ash near orchid, AO-Orch – orchid near ash, and ONA – orchid, no ash. Identical names followed by the same clade (C) number signify ASVs that are within the same ML clade (<0.01 evolutionary distance, and likely belong to a single organism).

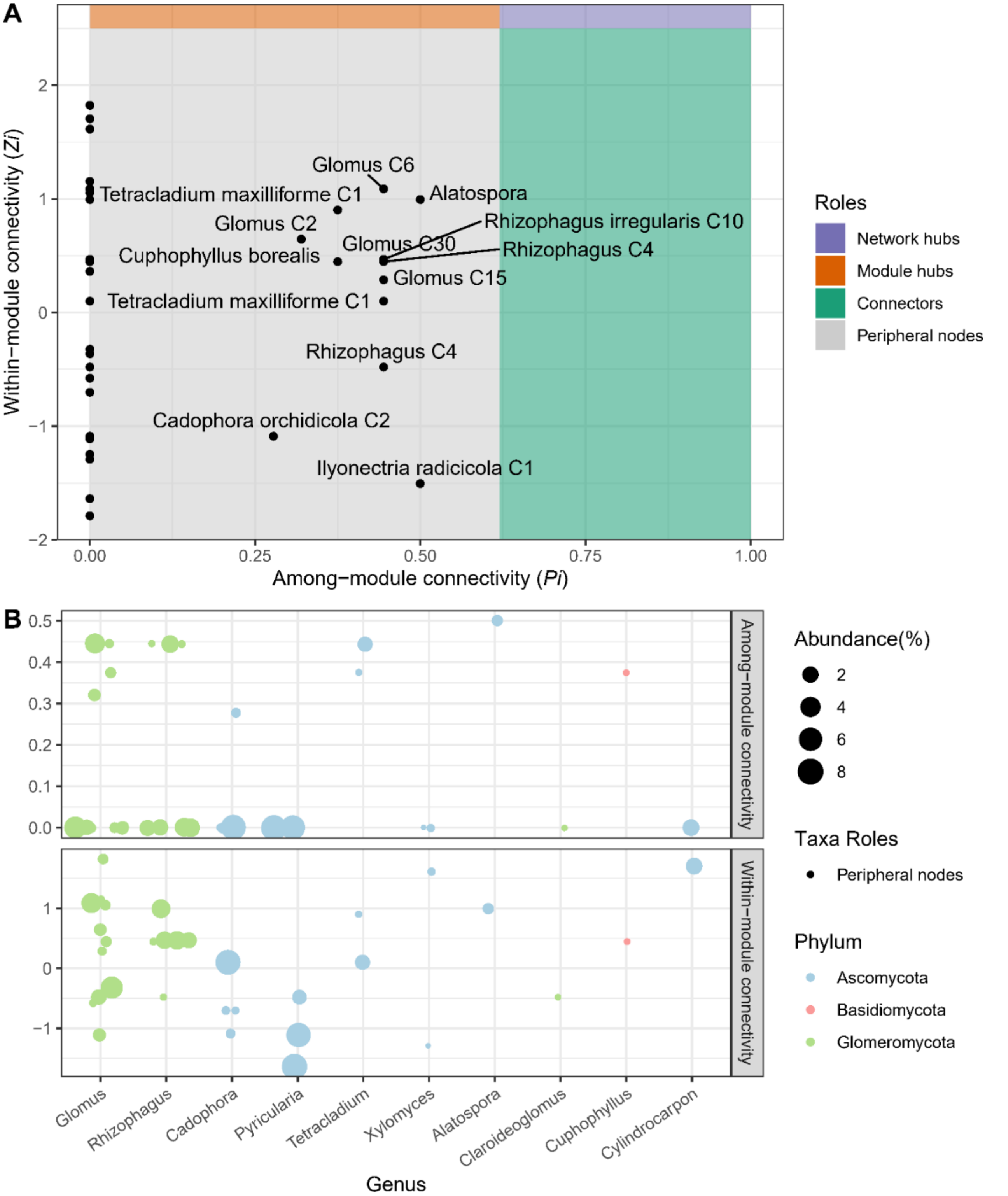

Supplementary Figure 6. Within-module (*Zi*) and among-module (*Pi*) connectivity plot of surface-sterilized (SS) ITS2 ASVs shared between black ash (*Fraxinus nigra*) and showy lady's slipper orchid (*Cypripedium reginae*). (A) *Zi-Pi* plot showing the distribution of ASVs based on topological roles, with labeled points highlighting ASVs with high relative *Zi* and *Pi* scores and (B) distribution

of all genera within the network. Abundance calculated within the 50 ASVs found in the trimmed SS dataset.

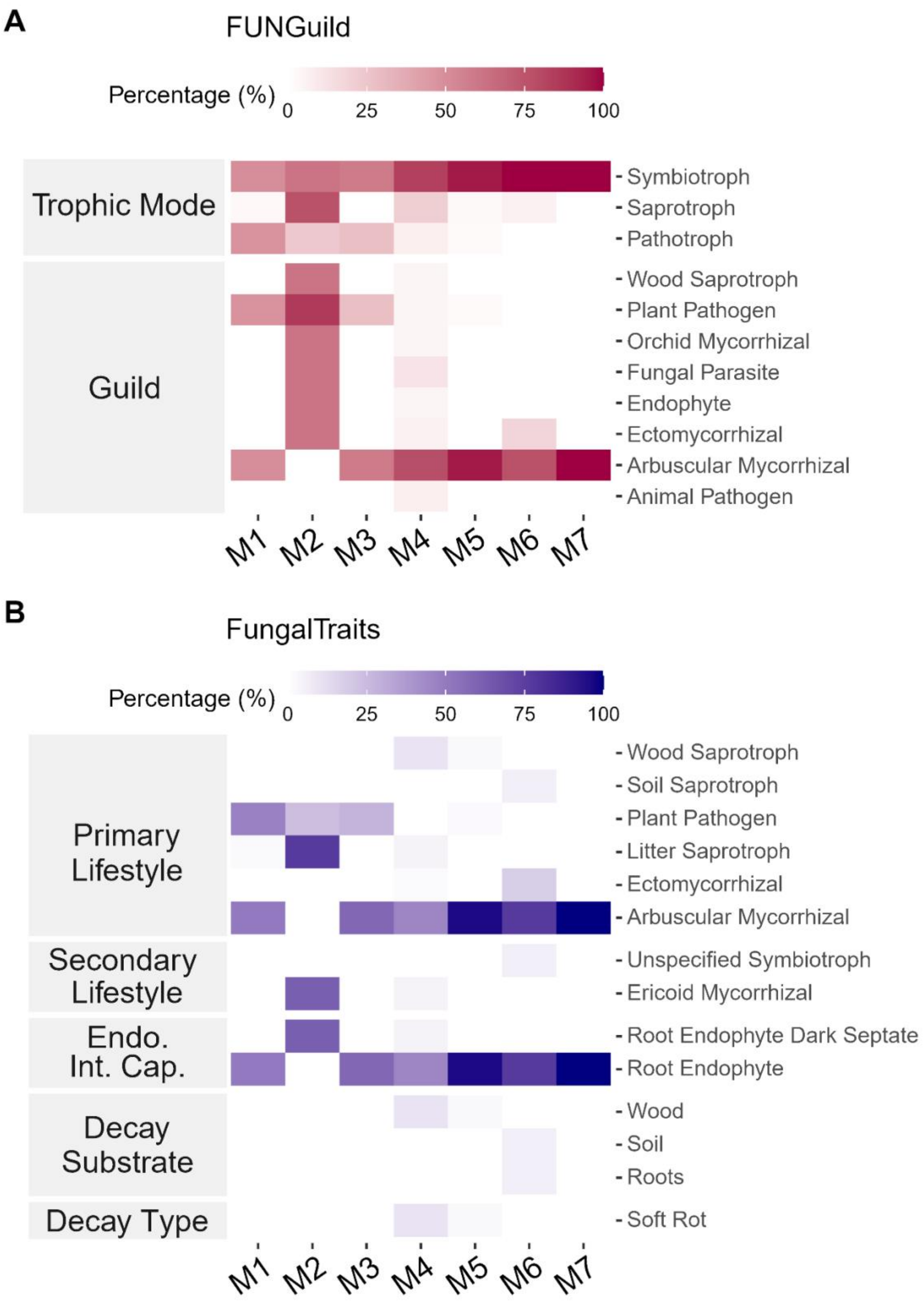
A
FUNGuild
Percentage (%)
0
25
50
75
100
Trophic Mode
Guild
Symbiotroph
Saprotroph
Pathotroph
Wood Saprotroph
Plant Pathogen
Orchid Mycorrhizal
Fungal Parasite
Endophyte
Ectomycorrhizal
Arbuscular Mycorrhizal
Animal Pathogen
M1
M2
M3
M4
M5
M6
M7
B
FungalTraits
Percentage (%)
0
25
50
75
100
Primary Lifestyle
Secondary Lifestyle
Endo. Int. Cap.
Decay Substrate
Decay Type
Wood Saprotroph
Soil Saprotroph
Plant Pathogen
Litter Saprotroph
Ectomycorrhizal
Arbuscular Mycorrhizal
Unspecified Symbiotroph
Ericoid Mycorrhizal
Root Endophyte Dark Septate
Root Endophyte
Wood
Soil
Roots
Soft Rot
M1
M2
M3
M4
M5
M6
M7

Supplementary Figure 7. Functional traits of weighted relative abundance taxa within each module (1-7) of the network analysis. Traits determined by (A) FUNGuild, and (B) FungalTraits. Endo. Int. Cap. – Endophytic Interaction Capability.

Supplementary Table 1. Sample metadata and grouping variables in analysis. Each sample was sequenced using ITS2 specific primers.

Sample	Presence	Plant	Location	Surface-Sterilized	Batch
ANOGH5	ANO	Ash	Humber Gorge	No	1
ANOGH3	ANO	Ash	Humber Gorge	No	1
ANOGH2	ANO	Ash	Humber Gorge	No	2
ANOHG1	ANO	Ash	Humber Gorge	Yes	1
ANOGH4	ANO	Ash	Humber Gorge	Yes	2
ANO5	ANO	Ash	Humber Village	No	1
ANO4	ANO	Ash	Humber Village	No	2
ANO1	ANO	Ash	Humber Village	Yes	1
ANO3	ANO	Ash	Humber Village	Yes	1
ANO2	ANO	Ash	Humber Village	Yes	2
ANOP2	ANO	Ash	Pasadena	No	1
ANOP1	ANO	Ash	Pasadena	No	2
ANOP3	ANO	Ash	Pasadena	No	2
ANOP5	ANO	Ash	Pasadena	Yes	1
ANOP6	ANO	Ash	Pasadena	Yes	2
AO10	AO-Ash	Ash	Humber Village	No	1
AO12	AO-Ash	Ash	Humber Village	No	2
AO4	AO-Ash	Ash	Humber Village	Yes	1
AO2	AO-Ash	Ash	Humber Village	Yes	2
AO9	AO-Ash	Ash	Humber Village	Yes	2
AOP1	AO-Ash	Ash	Pasadena	No	1
AOP2	AO-Ash	Ash	Pasadena	Yes	2
AOS3	AO-Ash	Ash	Steady Brook	No	1
AOS2	AO-Ash	Ash	Steady Brook	No	2
AOS4	AO-Ash	Ash	Steady Brook	No	2
AOS1	AO-Ash	Ash	Steady Brook	Yes	1
AOS5	AO-Ash	Ash	Steady Brook	Yes	1
AO10	AO-Orch	Orch	Humber Village	No	2
AO12	AO-Orch	Orch	Humber Village	No	1
AO4	AO-Orch	Orch	Humber Village	Yes	2
AO2	AO-Orch	Orch	Humber Village	Yes	1
AO9	AO-Orch	Orch	Humber Village	Yes	1
AOP1	AO-Orch	Orch	Pasadena	No	2
AOP2	AO-Orch	Orch	Pasadena	Yes	1
AOS3	AO-Orch	Orch	Steady Brook	No	2
AOS2	AO-Orch	Orch	Steady Brook	No	1
AOS4	AO-Orch	Orch	Steady Brook	No	1

AOS1	AO-Orch	Orch	Steady Brook	Yes	2
AOS5	AO-Orch	Orch	Steady Brook	Yes	2
ONAC1	ONA	Orch	Corner Brook	No	1
ONAC5	ONA	Orch	Corner Brook	No	1
ONAC3	ONA	Orch	Corner Brook	Yes	1
ONAC2	ONA	Orch	Corner Brook	Yes	2
ONAC4	ONA	Orch	Corner Brook	Yes	2
ONA6	ONA	Orch	Humber Village	No	1
ONA5	ONA	Orch	Humber Village	No	2
ONA4	ONA	Orch	Humber Village	Yes	1
ONA3	ONA	Orch	Humber Village	Yes	2
ONA7*	ONA	Orch	Humber Village	Yes	2
ONAU2	ONA	Orch	University	No	1
ONAU3	ONA	Orch	University	No	2
ONAU5*	ONA	Orch	University	No	2
ONAU1*	ONA	Orch	University	No	2
ONAU4	ONA	Orch	University	Yes	1

* samples were filtered prior to analysis due to low read count.

Supplementary Table 2. DADA2 settings, read retention after filtration and merging steps, and manual filtration steps for each primer. Singleton cutoff parameters removed amplified sequence variants (ASVs) that had fewer than 10 reads across all samples.

**Primer**	**ITS2**
**Settings**	
TruncQ	2
Max Errors	2,2
Min. Len.	50
TruncLen	-
Merged Length Trim	-
Chimera Detection	Consensus
**dada2**	
Input	1,304,351
Filtered	868,546
Denoised	858,984
Merged	798,665
Tabled	798,665
Non-Chimera	789,387
Final Reads	678,105
ASVs	1,114
**Manual Filtration**	
Rel. Abund. Cutoff	0.03%
Singleton Cutoff	10 reads
Total Reads	640,427
Total ASVs	1,012
**Target Reads**	**630,513**
**Target ASVs**	**993**

Supplementary Table 3. Alpha diversity measures of ITS2 amplified sequence variants (ASVs) from surface-sterilized and non-surface-sterilized black ash (*Fraxinus nigra*) and showy lady's slipper (*Cypripedium reginae*) roots. Orchid-ash presence groups: ANO – ash, no orchid; AO-Ash – ash near orchid, AO-Orch – orchid near ash, and ONA – orchid, no ash. DMRT – Duncan's Mean Range Test.

		**Surface-sterilized**			**Non-Surface-Sterilized**		
**Measure**	**Presence**	**N**	**mean ± SD**	**DMRT**	**N**	**mean ± SD**	**DMRT**
**ITS2**							
**Observed**	ANO	7	86.57 ± 11.89	a	8	92.13 ± 22.11	a
	AO-Ash	6	73 ± 22.81	a	6	109.83 ± 31.97	a
	AO-Orch	5	20.4 ± 14.81	b	6	21.5 ± 20.4	b
	ONA	6	16.33 ± 14.26	b	6	19.33 ± 20.1	b
**Shannon (H)**	ANO	7	3.33 ± 0.3	a	8	3.25 ± 0.51	a
	AO-Ash	6	3.17 ± 0.63	ab	6	3.68 ± 0.55	a
	AO-Orch	5	2.52 ± 0.65	bc	6	2.28 ± 0.6	b
	ONA	6	2.21 ± 0.86	c	6	2.16 ± 1.04	b
**Simpson (D)**	ANO	7	0.92 ± 0.03	a	8	0.9 ± 0.09	a
	AO-Ash	6	0.9 ± 0.06	ab	6	0.93 ± 0.08	a
	AO-Orch	5	0.88 ± 0.05	ab	6	0.83 ± 0.09	a
	ONA	6	0.83 ± 0.1	b	6	0.78 ± 0.21	a
**InvSimpson (1/D)**	ANO	7	15.72 ± 7.78	a	8	15.08 ± 8.49	ab
	AO-Ash	6	16.97 ± 14.51	a	6	27.33 ± 16.99	a
	AO-Orch	5	11.45 ± 8.43	a	6	8.41 ± 6.31	b
	ONA	6	9.59 ± 8.44	a	6	10.34 ± 12.32	b
**Fisher**	ANO	7	11.38 ± 1.59	a	8	12.19 ± 2.9	a
	AO-Ash	6	9.76 ± 3.09	a	6	15.14 ± 4.29	a
	AO-Orch	5	3.8 ± 2.82	b	6	3.67 ± 2.98	b
	ONA	6	3.19 ± 2.66	b	6	3.55 ± 3.88	b

Supplementary Table S4. Taxa shared between surface-sterilized roots of showy lady's slipper orchid (*Cypripedium reginae*) and black ash (*Fraxinus nigra*) growing within 15 m of one another (Orchid and Ash), and taxa unique to surface-sterilized roots of orchid or ash, based on identifications of ITS2 metabarcoding data. Symbols denote likely trophic modes for shared orchid and ash taxa based on FUNGuild and FungalTraits: * symbiotroph, † saprotroph, ‡ pathotroph.

**Orchid and Ash**	**Orchid**	**Ash**
Alatospora†	*Cadophora* C2	*Agrocybe erebia*
Cadophora orchidicola C1*†	*Ceratobasidium albasitensis*	*Alatospora acuminata*
Cadophora orchidicola C2*†	*Hyaloscypha finlandica*	*Cadophora* C1
*Claroideoglomus claroideum**	*Glomus sp Att565 7* C1	*Cadophora melinii*
Cuphophyllus borealis†	*Glomus versiforme* C1	*Calyptella capula*
Cylindrotrichum oligospermum C9†	*Inocybe geophylla*	*Ceratobasidium*
Dactylonectria macrodidyma C1‡	*Inocybe griseolilacina* C1	*Cistella*
Dactylonectria pauciseptata‡	*Inocybe sindonia* C1	*Cistella caricis*
Dominikia C5*	*Laccaria laccata var pallidifolia*	*Cladophialophora*
Glomus C1*	*Myrmecocystis microspora*	*Clathrosporium intricatum*
Glomus C2*	*Sebacina incrustans* C16	*Coprinopsis*
Glomus C3*	*Sebacina incrustans* C8	*Cryptosporiopsis radicicola*
Glomus C6*	*Tomentella* C9	*Dimorphospora foliicola*
Glomus C15*	*Xylomyces aquaticus* C2	*Eocronartium*
Glomus C29*	*Zalerion arboricola*	*Exophiala*
Glomus C30*	unknown Eurotiales	*Exophiala equina*
Glomus macrocarpum C1*	unknown Helotiales	*Exophiala opportunistica*
Glomus macrocarpum C2*	unknown Eurotiomycetes	*Exophiala* sp KL 2011f
Glomus macrocarpum C5*	unknown Ascomycota	*Glutinoglossum heptaseptatum*
Glomus macrocarpum C11*		*Herpotrichia juniperi*
Glomus sp 3 SUN 2011 C2*		*Hymenoscyphus epiphyllus*
Glomus sp 3 SUN 2011 C11*		*Idriella*
Ilyonectria radicicola C1‡		*Ilyonectria robusta*
*Inocybe ochroalba**		*Minimelanolocus obscurus*
Leptosphaeria‡†		*Mirandina breviphora*
Pyricularia C1‡		*Neonectria*
Pyricularia C2‡		*Orbilia*
Remispora stellata C5†		*Paraconiothyrium*
Rhizophagus C4*		*Phaeomoniella prunicola*
Rhizophagus intraradices C2*		*Phomopsis columnaris*
Rhizophagus intraradices C3*		*Podospora*
Rhizophagus intraradices C5*		*Podospora intestinacea*
Rhizophagus irregularis C2*		*Pyronemataceae* C2
Rhizophagus irregularis C10*		*Remispora stellata* C6

Sebacina incrustans C5*		*Remispora stellata* C7
Spirosphaera cupreorufescens C1†		*Remispora/Cirrenalia* C8
Tetracladium maxilliforme C1†		*Rhexocercosporidium panacis* C10
Tetracladium sp WMM_2012a C1†		*Rhinocladiella sp YH 2009a*
Tomentella C4*		*Sebacina incrustans* C1
Tomentella C24*		*Sebacina incrustans* C3
Tomentella galzinii C4*		*Sebacina incrustans* C4
Varicosporium†		*Sebacina incrustans* C12
Xylomyces aquaticus C1†‡		*Sebacina incrustans* C15
unknown Helotiales		*Sebacina incrustans* C17
unknown Glomeromycota*		*Sebacina incrustans* C9
unknown Ascomycota		*Sebacina vermifera* C3
		Septobasidium
		Spirosphaera cupreorufescens
		Tetracladium C1
		Tetracladium C4
		Tetracladium furcatum C1
		Tetracladium marchalianum
		Trichocladium opacum
		Veronaea botryosa
		Zalerion varium
		Zopfiella
		unknown Halosphaeriaceae
		unknown Helotiaceae
		unknown Orbiliaceae
		unknown Thelephoraceae
		unknown Auriculariales *inc. sed.*
		unknown Diaporthales
		unknown Helotiales
		unknown Hypocreales
		unknown Leotiales
		unknown Pleosporales
		unknown Sebacinales
		unknown Agaricales
		unknown Microascales
		unknown Chaetosphaeriales
		unknown Agaricomycetes inc. sed.
		unknown Dothideomycetes
		unknown Sordariomycetes
		unknown Leotiomycetes
		unknown Basidiomycota

		unknown Ascomycota
